# Trait‐Mediated Seed Predation and Dispersal Are Regulated by Seed Abundance for Sympatric Tree Species in the Qinling Mountains, China

**DOI:** 10.1002/ece3.74453

**Published:** 2026-10-04

**Authors:** Jing Wang, Ge Han, Bo Zhang, Ning Han, Tuo Feng, Jidong Zhao, Xiaolei An, Xiang Hou, Gang Chang

**Affiliations:** ^1^ Shaanxi Key Laboratory of Qinling Ecological Security Shaanxi Institute of Zoology Xi'an China; ^2^ Microbiology Institute of Shaanxi Xi'an China; ^3^ School of Biomedicine and Food Engineering Shangluo University Shangluo China

**Keywords:** forest rodents, seasonality, seed abundance, seed dispersal effectiveness, seed traits, sympatric species

## Abstract

Seed traits and seed abundance both affect rodent‐mediated seed predation and dispersal. However, many previous studies have investigated these two factors independently; few have explored whether the effects of seed traits on rodent‐mediated seed predation and dispersal vary with background seed abundance and across seasons. Here we investigated three sympatric Fagaceae species ( *Quercus variabilis* , *Quercus aliena*, and *Quercus glandulifera*) with contrasting seed traits to determine how seed traits, seed abundance and seasonality shaped rodent‐mediated seed dispersal and predation in the Qinling Mountains, China. Analysis of data from autumn 2016–2021 and spring 2022–2024 revealed clear rodent preferences among co‐occurring seed species with contrasting traits. *Q. variabilis* seeds were harvested more rapidly and cached more frequently than those of *Q. aliena* and *Q. glandulifera* regardless of seed abundance or seasonal variation, whereas *Q. glandulifera* seeds were harvested most slowly, consumed most frequently, particularly in situ, and rarely cached. These patterns suggested that seed trait‐mediated seed selection by rodents may play an important role in shaping masting effects on rodent‐mediated seed predation and dispersal. We found that seed abundance also affected seed fates: lower seed abundance was associated with faster seed harvesting, whereas higher seed abundance was associated with greater seed consumption and lower seed caching. These patterns supported both the predator satiation and predator dispersal hypotheses, indicating that the two mechanisms can operate concurrently. Seasonal variation also affected seed fates. Caching was significantly more frequent in autumn, whereas consumption dominated in spring, when few seeds were cached. Our findings provide broader insights into how seed traits, seed abundance and seasonality shape rodent‐mediated seed predation and dispersal, while providing a practical basis for promoting the natural regeneration of Fagaceae broad‐leaved forests, conserving oak germplasm, and guiding artificial tending and afforestation in the Qinling Mountains.

## Graphical abstract

We examined the effects of seed traits, seed abundance and seasonality on rodent‐mediated seed predation and dispersal of three sympatric oaks in the Qinling Mountains, China. Rodents consistently preferred *Quercus variabilis* seeds for caching, while *Quercus glandulifera* seeds were mostly consumed in situ. Low seed abundance accelerated harvesting, while high abundance increased consumption and reduced caching. Seasonal variation also affected seed fates, caching was significantly more frequent in autumn, whereas consumption dominated in spring, when few seeds were cached. These findings provide crucial insights into the natural regeneration and conservation of oak forests.

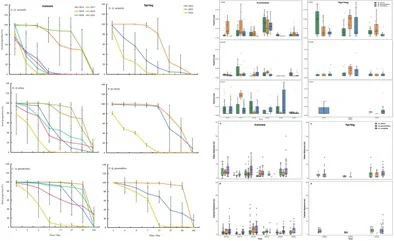

## 1 Introduction

The interaction between rodents and seed‐bearing plants is a vital ecological mutualism (Chang and Zhang 2014; Han et al. 2025). While rodents rely on seeds as a source of food either for immediate or deferred consumption, through caching (Wang and Chen 2009), plants must attempt to balance the appeal of their seeds against the risk of too great a proportion being predated and destroyed (Zhang, Shi, et al. 2016; Zhang, Yan, et al. 2016). Rodent‐mediated zoochorous seed dispersal services thus present a shared and potentially limiting resource that can result in exploitation competition among plant species (Xiao et al. 2022). Nevertheless, how co‐existing plant species sequester a share of this service to optimize their seed dispersal remains a major unresolved question in community ecology (Silvertown 2004).

Zoochorous plant species produce seeds with traits that appeal to rodents (Yang et al. 2012), moderated by defenses to reduce rates of seed destruction through predation (Chen et al. 2023). Furthermore, rodent species each exhibit preferences for seeds with contrasting traits (e.g., Cao et al. 2016; Yang et al. 2019; Wang et al. 2023). Previous studies have established that larger seeds with higher nutritional value, higher tannin content and a thicker seed coat are often dispersed and cached more often by rodents, while smaller seeds with lower nutritional value, a lower tannin content and a thinner seed coat are typically consumed immediately in situ, resulting in lower seed‐dispersal success (e.g., Vander Wall 2010; Vander Wall and Beck 2012; Zhang, Shi, et al. 2016; Zhang, Yan, et al. 2016; Sundaram et al. 2016; Jiang and Yi 2025). Seed traits and mast seeding therefore both contribute to relative rates of seed dispersal and predation by rodents (Li and Zhang 2007; Schnurr et al. 2002). Furthermore, rodent seed dispersal and predation strategies may be affected by the relative appeal and abundance (i.e., availability) of seeds from other sympatric tree species competing for rodent‐mediated dispersal services (Wang 2020). In seed communities, this can thus result in indirect seed‐seed interactions among sympatric plant species (e.g., Lai et al. 2014; Zhang, Shi, et al. 2016; Zhang, Yan, et al. 2016; Bogdziewicz et al. 2019; Yang et al. 2019). Because no two species can occupy exactly the same niche hypervolume (Gause's law; Meszéna et al. 2006), “competitive exclusion” causes species to adopt subtly different alternate life‐history strategies, where each partitions contested resources to occupy a distinct “realized niche” (Kitajima and Poorter 2008). Consequently, sympatric spermatophyte species with different seed traits may have different mutualistic interactions or different fitness consequences for seed dispersal.

Seed masting rates can contribute substantially to seed dispersal and predation rates (e.g., Lichti et al. 2014; Qiu et al. 2023; Wang et al. 2023). Masting rates vary interannually, often linked to prevailing weather conditions (Bogdziewicz et al. 2024), and can differ consistently among species (Yang et al. 2019), even closely related species (Dale et al. 2021). Masting rate variation is common among many perennials that synchronously produce large seed crops at irregular interannual intervals (e.g., Lichti et al. 2014; Pearse et al. 2016; Yang et al. 2019; Xiao and Huang 2020; Yu et al. 2020). A number of hypotheses have been proposed to explain this phenomenon (e.g., Koenig 2021; Bogdziewicz et al. 2024), however, the most widely accepted hypotheses explaining mast seeding are the predator satiation hypothesis (e.g., Fletcher et al. 2010; Bogdziewicz et al. 2018; Zwolak et al. 2022) and the predator dispersal hypothesis (e.g., Jansen et al. 2004; Zwolak et al. 2016; Yang et al. 2019). Both hypotheses propose that seed dispersal is generally regulated by a combination of annual seed production and rodent abundance (e.g., Schnurr et al. 2002; Jansen et al. 2004; Li and Zhang 2007; Yi et al. 2011; Yang et al. 2019). Thus, per capita seed availability as the product of annual seed production in relation to annual rodent abundance can provide a framework for investigating the regulation of seed dispersal effectiveness (e.g., Schupp et al. 2010; Wang et al. 2023; Xiao et al. 2013). Furthermore, seasonal food availability may interact with masting, because abundant food resources for rodents in autumn resemble masting conditions, whereas seed scarcity in spring resembles non‐masting conditions (Chang and Tai 2011).

Rodents either harvest seeds as a source of nutrition or carry seeds to more distant locations to scatter‐hoard them as a food cache for later consumption (e.g., Vander Wall 2010; Wang et al. 2013; Yi et al. 2016; Lichti et al. 2017). However, a proportion of these scatter‐hoarded seeds will be forgotten and remain unused, and these seeds may go on to germinate successfully (e.g., Lichti et al. 2017; Hou et al. 2021; Zwolak et al. 2024). Nevertheless, there is a lack of systematic research on whether there are differences in rodent dispersal and predation rates between seasons.

Seed dispersal effectiveness (SDE) can be used to synthesize the quantity and quality of animal‐mediated dispersal each plant species receives (e.g., Schupp 1993; Schupp et al. 2010; Leeuwen et al. 2022; Nevo et al. 2023), providing a framework for evaluating zoochorous seed dispersal. Ultimately, each plant species in a community will seek to match or exceed the share of zoochorous seed dispersal it can sequester relative to its niche competitors. Nevertheless, the differentiation of SDE among interacting sympatric plant species competing for scatter‐hoarding rodent seed dispersal services remains to be fully resolved (e.g., Green et al. 2022; Nevo et al. 2023).

In this study, we addressed this knowledge gap by tracking the annual seed crop production and seed dispersal and predation fates of three sympatric congeneric tree species ( *Quercus variabilis* , *Quercus aliena*, and *Quercus glandulifera*) in autumn from 2016 to 2021 and in spring from 2022 to 2024 in the Qinling Mountains, Shaanxi Province, China. These tree species dominate the forest at the study site, and their seeds are an important food source for the local rodent community (Zhang et al. 2019; Wang et al. 2023). We addressed the following questions: (1) When rodents encounter the three co‐occurring *Quercus* seed species with contrasting traits, do they show clear preferences? (2) If so, which seed species has a stronger competitive advantage for seed dispersal? (3) Do seasonal and annual changes in seed abundance affect rodents' seed‐selection strategies?

## 2 Materials and Methods

### 2.1 Study Site and Species

This study was conducted in the Foping National Nature Reserve (33°33′ N–33°46′ N, 107°41′ E–107°55′ E), located on the southern slopes of the Qinling Mountains in Shaanxi Province, Central China (Figure 1). This region lies at the northern edge of the transition from the north subtropical climate zone to the warm temperate zone, with a mean annual temperature and precipitation of 11.5°C and 924 mm, respectively (Zhang et al. 2019; Wang et al. 2021). The study site covered 3 km^2^, with a mean elevation of 1200 m. Here, the deciduous broad‐leaved forest was dominated by *Q. variabilis* , *Q. aliena*, and *Q. glandulifera*, along with the common occurrence of *Pinus tabuliformis* , *Cyclobalanopsis glauca*, *inter alia*. Twenty‐eight rodent species have been recorded in this forest area, of which *Niviventer confucianus* and *Apodemus draco* occur most abundantly (e.g., Chang et al. 2012; Wang et al. 2023).

**FIGURE 1:**
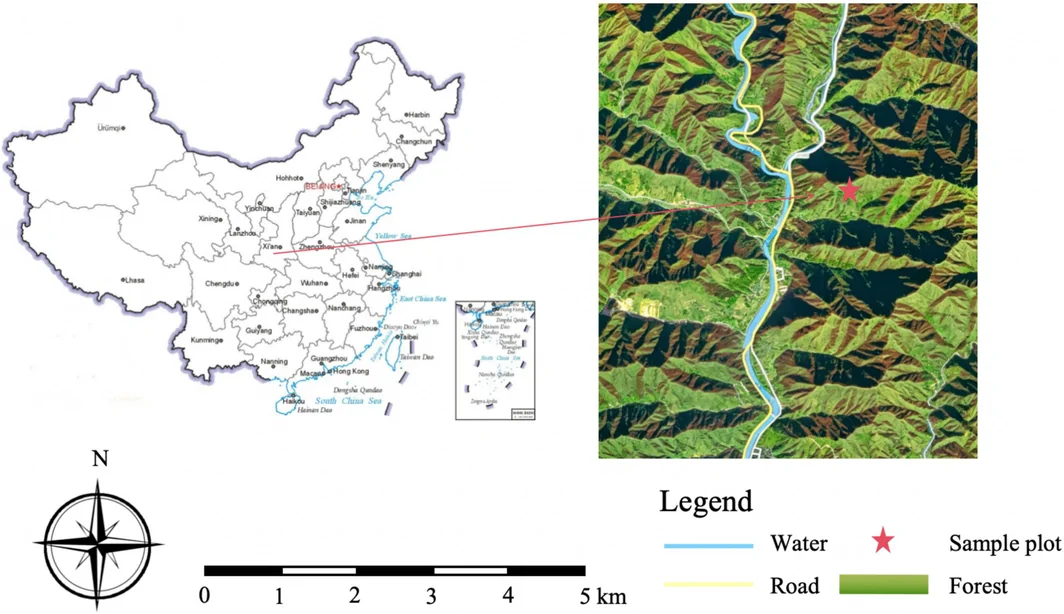
Location of the study site within Foping National Nature Reserve in the Qinling Mountains, Shaanxi Province, China, with the inset showing the location of the study area within the reserve.

### 2.2 Seed and Rodent Abundance

To estimate annual seed crop from 2016 to 2021, eight mature, similarly sized individuals of *Q. variabilis* , *Q. aliena*, and *Q. glandulifera* were randomly selected, and four seed rain collection traps were set under the canopy of each individually numbered tree along compass bearings (east, south, west, north). Each 1 m^2^ trap was set 1.5 m above the ground to prevent seeds from bouncing out of the frame or being eaten by animals (e.g., Zhang et al. 2019; Wang et al. 2021, 2023). Seed rain collections were conducted weekly from 20th August (when seed fall commenced) to 24th November (when seed fall concluded) each year. Collected seeds were sorted according to four types (intact seed, larvae‐infested seed, abortive seeds, and empty cupules). Annual seed crop was then calculated as the sum of intact and infested seeds per square meter (N/m^2^).

We also measured natural seed morphological and chemical defense traits (Table 1) and tested differences between the three *Quercus* spp. as an indicator of likely differences in appeal to the rodent disperser community.

**TABLE 1:** Physical and chemical traits of *Quercus variabilis* , *Quercus aliena*, and *Quercus glandulifera* seeds.

Seed traits	*Q. variabilis*	*Q. aliena*	*Q. glandulifera*
Diameter^a^ (mm)	15.73 ± 0.080	11.71 ± 0.034	9.96 ± 0.042
Length^a^ (mm)	18.52 ± 0.057	15.36 ± 0.051	15.03 ± 0.048
Pericarp thickness^a^ (mm)	0.51 ± 0.003	0.35 ± 0.002	0.26 ± 0.002
Fresh weight^a^ (g)	3.05 ± 0.035	1.16 ± 0.012	0.72 ± 0.010
Crude protein^b^ (%)	4.25	5.10	3.91
Crude fat^b^ (%)	3.67	1.68	1.98
Crude starch^b^ (%)	41.79	38.12	40.10
Crude fiber^b^ (%)	2.04	1.96	1.78
Tannin^b^ (%)	7.36	8.34	8.76

^a^ Values are Mean ± S.E., *N* = 160 for each species.

^b^ Seed chemical composition was determined by the Grain Quality Supervision and Testing Center, Ministry of Agriculture (No. K120041‐K120047). A subset of seeds from each species was mailed to the official testing institution for standardized component detection.

We used live traps (27.0 cm × 14.0 cm × 14.0 cm) to survey rodent abundance during the autumn (September and October) of each year from 2016 to 2021 (except 2018, due to lack of investigation). Traps were baited with peanuts and local dry leaves were provided as nesting materials to protect rodents from cold conditions. Traps were concealed in vegetation to prevent interference from potential predators. Trapping was conducted over 10 plots for three consecutive days. Within each plot, we randomly selected three transect lines along which 20 traps were set 10 m apart along, with transect lines spaced at least 15 m apart. (e.g., Wang et al. 2017, 2021). We recorded the species, sex and body mass of each individual rodent and the numbers captured per transect. We also marked each captured rodent with a red paint dot (Shaanxi paint industry co. LTD, Xian) to prevent reprocessing and recounting recaptures (Wang et al. 2017). All rodents were then released at the site of capture.

We used the relative abundance (RA_*i*_) and combined body mass (BM_*i*_) of rodents, across species, to calculate annual rodent availability (ARA).

$$\mathrm{ARA}=\sum_{i=1}^{k}{\mathrm{RA}}_{i}{\mathrm{BM}}_{i}$$

where *k* = total number of rodent species, RA_*i*_ = the relative abundance for each rodent species *i* (the number of individuals captured); BM_*i*_ = the average body mass for a given rodent species *i*.

Rodent‐mediated seed dispersal and predation rates can be affected by both seed abundance and rodent abundance; therefore we used per capita seed availability (PCSA) to quantify masting effects rather than only considering the abundance of each metric separately. PCSA was calculated based on annual seed crop (ASC, average seed crop per tree per year) and annual rodent availability (ARA, based on RA_*i*_ and BM_*i*_), (e.g., Yang et al. 2019; Wang et al. 2021, 2023). That is:

$$\text{PCSA}=\frac{\mathrm{ASC}}{\mathrm{ARA}}$$

### 2.3 Seed Dispersal Experiments

Seed dispersal experiments were conducted in the autumns of 2016 to 2021 and the springs of 2022 to 2024, using standard seed tagging techniques (see Wang et al. 2021, 2023). Briefly, a 0.7 mm diameter hole was drilled into the edge of each seed husk (pericarp) was attached using a portable electrical drill to which a small, coded plastic tag (2.5 × 3.5 cm, white) using a thin steel thread (0.2 mm in diameter and 15 cm in length) (Yang et al. 2019; Wang et al. 2023). When tagged seeds were removed, eaten or cached by rodents, remaining tags could easily be detected on the forest floor. Previous research has shown that this tagging technique does not tend to affect seed dispersal fates by rodents (e.g., Yang et al. 2019; Zhang et al. 2019; Wang et al. 2021).

We established 10 seed release stations, spaced 20 m apart, each with either 20 *Q. variabilis* , *Q. aliena*, and *Q. glandulifera* seeds. We then surveyed for released seeds fates after 1, 3, 5, 10, 17, 27, 50, and 162 days, and monitored the transposition of tagged seeds within a ≈30 m radius around each station. Seeds fates were defined as: (1) intact in situ (IIS); (2) eaten in situ (EIS); (3) eaten after removal (EAR); (4) scatter or larder cached in situ or after removal (cached on the surface soil or beneath leaf litter) (SLC). In addition, if a seed was eaten or cached, the distance between the seed's (or remaining tag's) final location and the associated release station was measured.

In this study, we used seed dispersal effectiveness (SDE) to compare the contribution of rodent‐mediated dispersal to the probability of seed survival until the following spring among the three *Quercus species* and under different levels of seed abundance. SDE was calculated as follows:

$$\begin{array}{c} \mathrm{SDE}=\frac{\text{The number of seeds removed}\;\mathrm{by}\;\text{rodents}}{\text{The number of seeds released}\;\mathrm{at}\;\text{the seed stations}}\;\\ \;\;\times \frac{\text{The number of seeds overwinter survival given removal}}{\text{The number of seeds removed}\;\mathrm{by}\;\text{rodents}\;}\times 100\%\\ =\frac{\text{The number of survived seeds after overwintering}}{\text{The number of released seeds}}\times 100\% \end{array}$$

where SDE represents the percentage of released seeds that survived until the following spring following rodent‐mediated dispersal (Yang et al. 2019).

### 2.4 Data Analysis

We employed binomial generalized linear mixed models (GLMMs) with a logit‐link function to examine seed dispersal fates (IIS, EIS, EAR, and SLC). Analyses were conducted separately for the autumn (2016–2021) and spring (2022–2024) datasets. We built two sets of independent GLMMs. The first set comprised year‐specific models, where *Quercus* species was specified as the fixed factor to detect interspecific differences in seed fate within each year. The second set included year as the fixed factor to evaluate overall inter‐annual variation in seed fate across the monitoring period. Seed fate was the dependent variable, and seed station was the random factor in all models. We also used GLMMs to test differences in natural seed crop (N/m^2^) among the three *Quercus* spp. across the 6 years (2016–2021), with seed crop as the dependent variable, year as a fixed effect, and sample tree as a random effect. Spearman correlation was used to analyze the relationship between seed availability (PCSA) and seed fates. We then used a general linear model (GLM) to test differences in dispersal distance among species and years. Finally, one‐way ANOVA was used to analyze differences in SDE among the three *Quercus* spp. and among years. For all analyses, means ± SD are reported, with significance set at *p* < 0.05 and high significance at *p* < 0.01.

## 3 Results

### 3.1 Rodent and Seed Abundance

From 2016 to 2021 (except 2018), we captured a total of 252 rodents from 11 species, including *Niviventer niviventer* (50%), *A. draco* (29.76%), *Cansumys canus* (2.78%), *Sciurotamias davidianus* (2.78%), *Musmusculus* (4.37%), *Rattus norvegicus* (3.97%), *Niviventer fulvescens* (2.78%), *Apodemus peninsulae* (1.59%), *Sciuridae* (0.79%), *Eothenomys Inez* (0.79%), and *Sorex araneus* (0.40%); that is, almost 80% of the rodent community was comprised of *N. niviventer* and *A. draco* . From these captures, we established that annual rodent availability (ARA) varied substantially among years, being highest in 2016 (81.05) and the lowest in 2020 (35.51) (Table 2).

**TABLE 2:** Annual rodent availability (ARA); annual seed crop of the three species (ASC); total annual seed crop of the three species (TASC); and the sum of per capita seed availability (PCSA) of the three species.

Year	Tree species	ASC	TASC	ARA	PCSA
2016	*Q. variabilis*	13.94 ± 2.58	69.50 ± 6.41	81.05	0.86
	*Q. aliena*	46.18 ± 5.48			
	*Q. glandulifera*	9.38 ± 1.40			
2017	*Q. variabilis*	21.66 ± 3.35	92.40 ± 11.77	54.27	1.70
	*Q. aliena*	56.12 ± 9.15			
	*Q. glandulifera*	14.63 ± 2.23			
2018	*Q. variabilis*	7.53 ± 1.57	66.39 ± 9.17	—	—
	*Q. aliena*	43.73 ± 7.09			
	*Q. glandulifera*	15.38 ± 2.12			
2019	*Q. variabilis*	11.00 ± 2.02	61.29 ± 5.74	68.04	0.90
	*Q. aliena*	42.40 ± 4.87			
	*Q. glandulifera*	7.89 ± 1.28			
2020	*Q. variabilis*	1.88 ± 0.43	46.75 ± 4.78	35.51	1.31
	*Q. aliena*	43.13 ± 4.53			
	*Q. glandulifera*	1.75 ± 0.41			
2021	*Q. variabilis*	1.22 ± 0.29	35.81 ± 5.91	44.59	0.80
	*Q. aliena*	28.06 ± 5.10			
	*Q. glandulifera*	6.53 ± 1.10			

*Note:* Seed production was calculated as the mean value from eight sample trees; rodent abundance was derived from live‐trapping surveys, with 1800 trap‐nights per survey year. No rodent trapping was conducted in 2018.

The annual seed crop (ASC) of *Q. variabilis* varied significantly across the 6 years study period (*p* < 0.01), ranging from a peak in 2017 (21.66 ± 3.35) to lows in 2020 (1.88 ± 0.43) and 2021 (1.22 ± 0.29); and the ASC *Q. glandulifera* also varied significantly among the 6 years (*p* < 0.01), the highest ASC of *Q. glandulifera* was in 2017 (14.63 ± 2.23) and 2018 (15.38 ± 2.12), and lowest in 2020 (1.75 ± 0.41) (Table 2). However, there were no significant differences in ASC for *Q. aliena* seeds among the 6 years (*p* = 0.355), and the highest ASC of *Q. aliena* was in 2017 (56.12 ± 9.15) and lowest in 2021 (28.06 ± 5.10) (Table 2). From combining ASC and ARA data, the sum of PCSA was highest in 2017 (1.70) and lowest in 2021 (0.80) (Table 2). Overall, the three sympatric *Quercus* spp. exhibited basically synchronized masting or non‐masting between years.

### 3.2 Effects of Seed Traits in Seed Dispersal and Predation

In autumn, the proportion of seeds found intact in situ (IIS) differed significantly among the three *Quercus* spp. in all 2016, 2017, 2020, and 2021 (all *p* < 0.01), 2018 (*p* < 0.05), but not in 2019 (*p* = 0.232) (Figure 2, Table S1). The proportions of seeds eaten in situ (EIS) differed significantly among the three *Quercus* spp. in 2016, 2018 and 2021 (all *p* < 0.01), but not in 2017 (*p* = 0.096), 2019 (*p* = 0.286), or 2020 (*p* = 0.158) (Figure 3, Table S1). The proportions of seeds eaten after removal (EAR) differed significantly among the three *Quercus* spp. in 2016, 2018, 2019, 2020, and 2021 (*p* < 0.01), but not in 2017 (*p* = 0.285) (Figure 3, Table S1). The proportions of seeds scatter‐ or larder‐cached (SLC) differed significantly among the three *Quercus* spp. in 2016, 2020, and 2021 (all *p* < 0.01), but not in 2017 (*p* = 0.302), 2018 (*p* = 0.114), or 2019 (*p* = 0.070) (Figure 3, Table S1). In contrast, eaten distances differed significantly among the three *Quercus* spp. only in autumn 2018 (*p* < 0.01), and cached distances did not differ significantly among the three *Quercus* spp. in any of the 6 years (all *p* > 0.05) (Figure 4). Overall, seed dispersal effectiveness (SDE) differed significantly among the three *Quercus* spp. in 2016, 2018, 2019, and 2020 (all *p* < 0.01), but not in 2017 or 2021 (all *p* > 0.05); *Q. variabilis* had the highest SDE in all years, followed by *Q. aliena*, whereas *Q. glandulifera* had the lowest.

**FIGURE 2:**
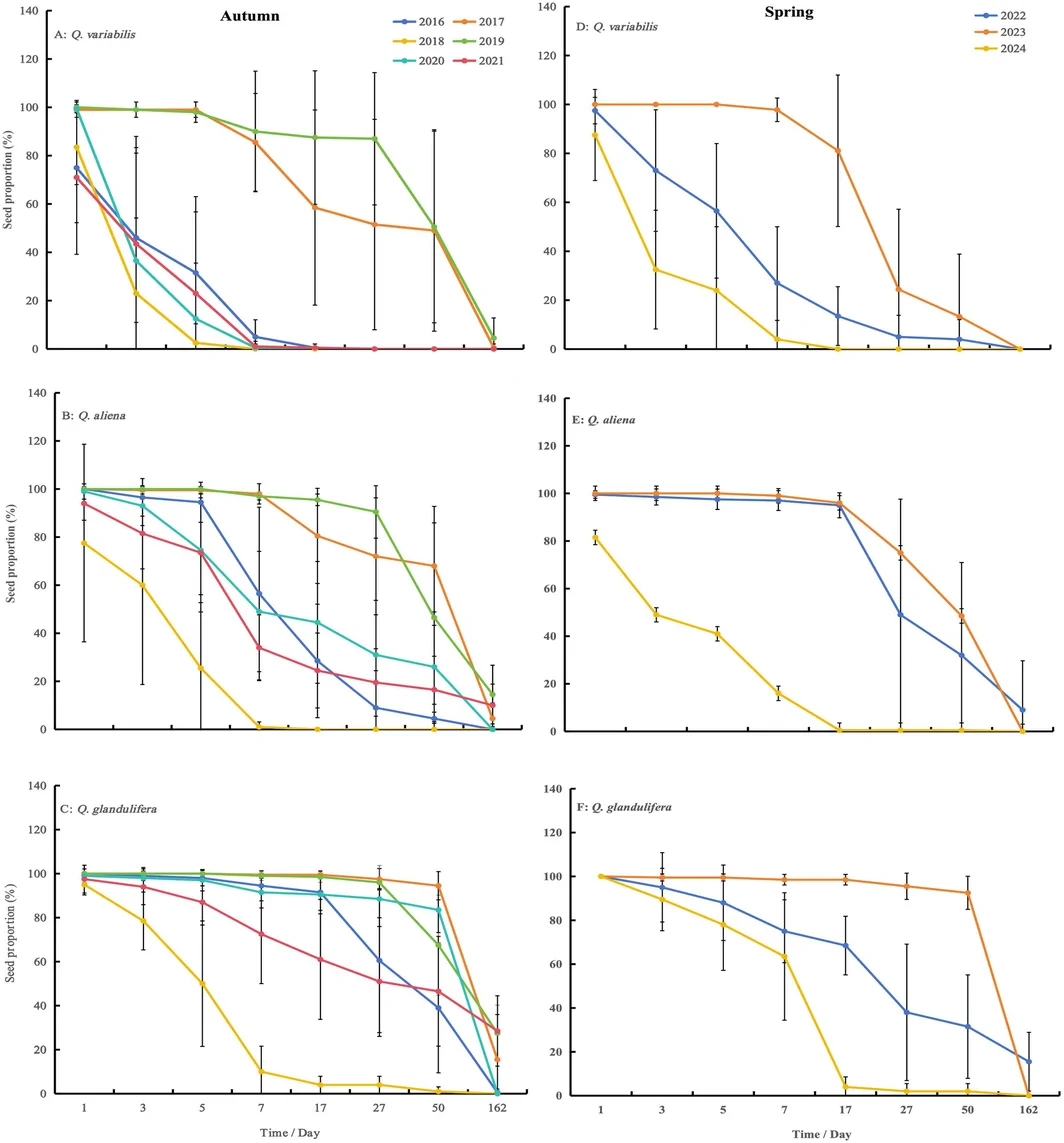
Seed harvest rates after placement of *Quercus variabilis* , *Quercus aliena*, and *Quercus glandulifera* seeds in autumn 2016–2021 (A–C) and spring 2022–2024 (D–F). Data are expressed as mean ± SD Sample size: *n* = 10 seed‐release stations; 20 seeds per species, 60 seeds in total per station.

**FIGURE 3:**
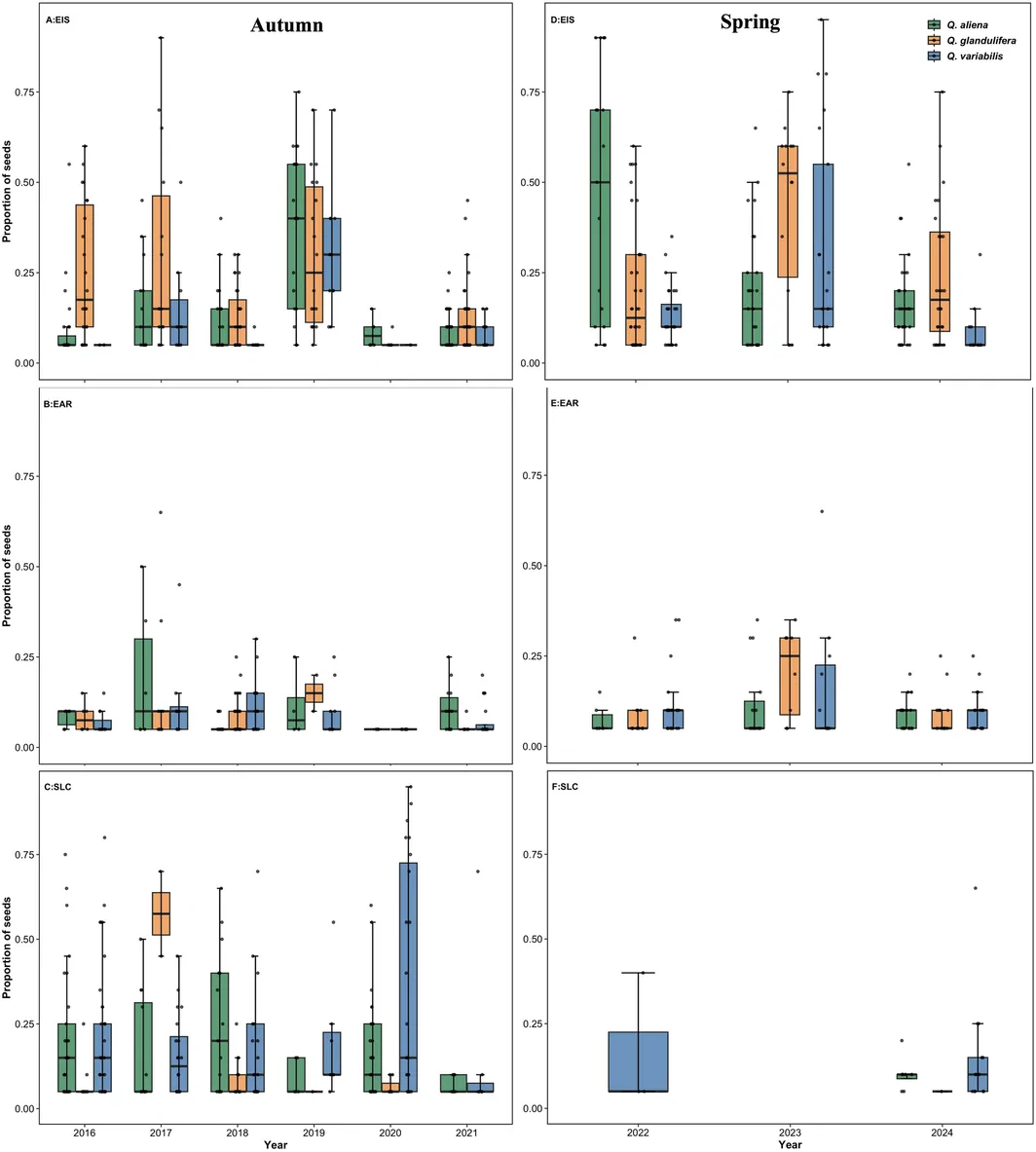
Seed dispersal and predation fates of *Quercus variabilis* , *Quercus aliena*, and *Quercus glandulifera* seeds in autumn 2016–2021 (A–C) and spring 2022–2024 (D–F). EAR, eaten after removal; EIS, eaten in situ; SLC, scatter‐ or larder‐cached. Data are expressed as mean ± SD Sample size: *n* = 10 seed‐release stations; 20 seeds per species, 60 seeds in total per station.

**FIGURE 4:**
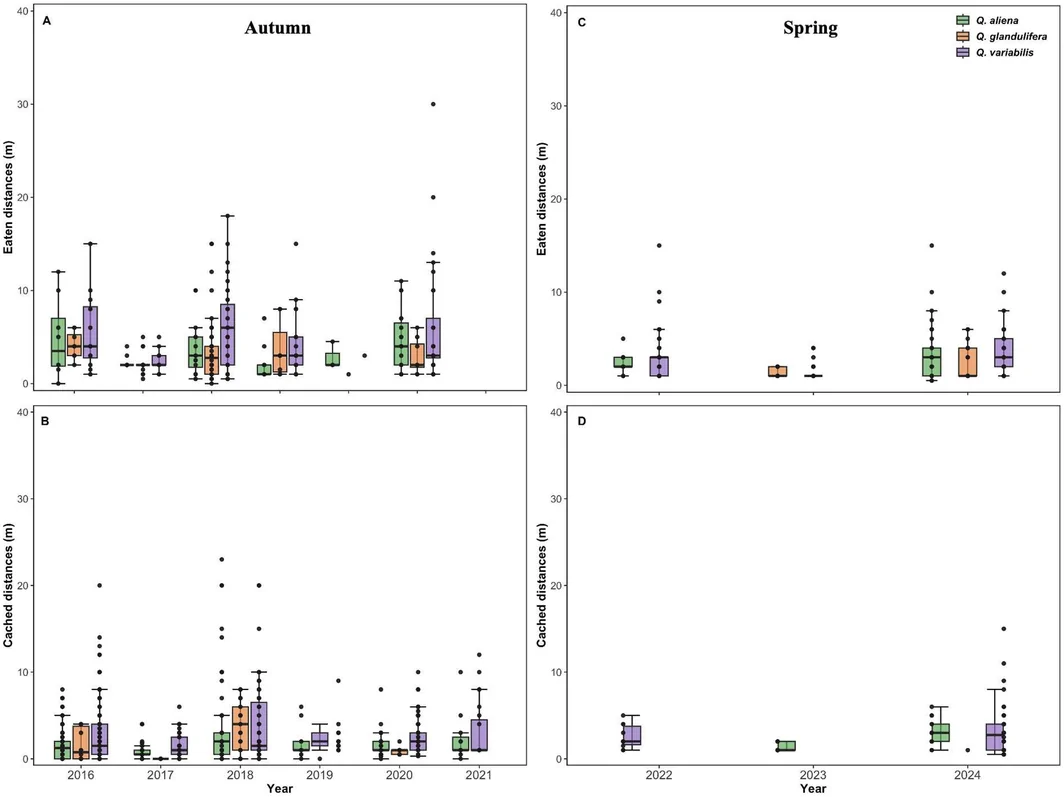
Distances to the final locations of eaten and cached *Quercus variabilis* , *Quercus aliena*, and *Quercus glandulifera* seeds in autumn 2016–2021 (A, B) and spring 2022–2024 (C, D). Sample size: *n* = 10 seed‐release stations; data include both eaten and cached distances of removed seeds.

In spring, IIS differed significantly among the three *Quercus* spp. in all 3 years (2022–2024) (all *p* < 0.01) (Figure 2, Table S2). EIS differed significantly among species in 2024 (*p* < 0.01), but not in 2022 (*p* = 0.113) or in 2023 (*p* = 0.826) (Figure 3, Table S2). EAR differed significantly among species in 2022 (*p* < 0.01), but not in 2023 (*p* = 0.987) or in 2024 (*p* = 0.249) (Figure 3, Table S2). SLC differed significantly among species in 2024 (*p* < 0.05), but not in 2022 (*p* = 1.000), and no seeds of any of the three *Quercus* spp. were cached in 2023 (Figure 3, Table S2). Neither eaten nor cached distances differed significantly among the three *Quercus* spp. in spring in any study year (all *p* > 0.05) (Figure 4). We detected no overall difference in SDE among the three *Quercus* spp., due to the low spring seed‐caching rates.

### 3.3 Effects of Annual and Inter‐Seasonal in Seed Dispersal and Predation

In autumn, IIS, EIS, SLC (all *p* < 0.01) and EAR (*p* < 0.05) for *Q. variabilis* differed significantly among the 6 years (2016–2021) (Figures 2 and 3, Table S3). For *Q. aliena*, IIS, EIS, and SLC differed significantly among the 6 years (all *p* < 0.01), whereas EAR (*p* = 0.101) did not differ significantly (Figures 2 and 3, Table S4). For *Q. glandulifera*, IIS, EAR, SLC (all *p* < 0.01) and EIS (*p* < 0.05) differed significantly among the 6 years (Figures 2 and 3, Table S5). Eaten and cached distances did not differ significantly among the 6 years for any of the three species (Figure 4). SDE differed significantly among years for *Q. variabilis* and *Q. aliena* (all *p* < 0.01), but not for *Q. glandulifera* (*p* > 0.05). Spearman correlation analysis indicated that only IIS for *Q. variabilis* and *Q. glandulifera* was positively correlated with summed PCSA (all *p* < 0.05); other correlations were not significant.

For the spring dataset, IIS and EIS for *Q. variabilis* differed significantly among the 3 years (2022–2024) (all *p* < 0.01), whereas EAR (*p* = 0.579) and SLC (*p* = 0.056) did not (Figures 2 and 3, Table S6). For *Q. aliena*, IIS, SLC (all *p* < 0.01) and EAR (*p* < 0.05) differed significantly among the 3 years, whereas EIS (*p* = 0.267) did not (Figures 2 and 3, Table S7). For *Q. glandulifera*, IIS and SLC differed significantly among the 3 years (all *p* < 0.01), whereas EIS (*p* = 0.857) and EAR (*p* = 0.603) did not (Figures 2 and 3, Table S8). The only significant difference in cached distance occurred for *Q. aliena* seeds in spring 2024 (*p* < 0.05), and no significant differences in eaten distance were detected for any *Quercus* spp. (Figure 4). We detected no overall difference in SDE among the three *Quercus* spp. across the 3 years, due to the low spring seed‐caching rates.

## 4 Discussion

In our study, we investigated three sympatric Fagaceae species ( *Q. variabilis* , *Q. aliena*, and *Q. glandulifera*) and examined how contrasting seed traits affected rodent‐mediated seed dispersal and predation in autumn 2016–2021 and spring 2022–2024. We found that rodents exhibited distinct foraging preferences among coexisting seeds with contrasting traits, indicating that seed‐trait filtering strongly influenced seed dispersal effectiveness. In addition, seed predation and dispersal fates differed markedly across years and seasons, indicating corresponding interannual and seasonal variation in rodents' seed‐foraging and caching strategies.

### 4.1 Seed Traits Mediated Effects

Seed traits, including seed mass, size, shell thickness, nutrient content, and tannin content, can affect rodent‐mediated seed predation and dispersal rates, with consequences for seed survival, seedling establishment, and plant regeneration (Hollander and Vander Wall 2004; Cao et al. 2016; Zhang, Shi, et al. 2016; Zhang, Yan, et al. 2016). Previous studies suggest that seeds with harder shells and higher nutritional value are cached more frequently and experience lower predation, whereas seeds with softer shells or lower nutritional value experience greater predation (Chang and Zhang 2014; Zhang et al. 2015; Zhang, Shi, et al. 2016; Zhang, Yan, et al. 2016). In our study, rodents exhibited distinct preferences among sympatric seeds with contrasting traits across both mast and non‐mast years (high vs. low seed abundance) and in both spring and autumn. *Q. variabilis* seeds, which were larger, more nutritious and lower in tannins, were preferred by rodents. They were harvested significantly faster than *Q. aliena* and *Q. glandulifera* seeds in almost every year, were cached most frequently, and experienced the lowest predation rates among the three *Quercus* spp. In contrast, *Q. glandulifera* seeds, which were smaller, less nutritious and higher in tannins, were harvested significantly more slowly than seeds of the other two species, were consumed most frequently (especially in situ), and were cached least frequently. *Q. aliena* and *Q. glandulifera* seeds differed little in nutritional composition, but *Q. aliena* seeds were heavier and larger. Although seed predation and dispersal fates differed little between these two species, rodents nevertheless showed a stronger preference for *Q. aliena* seeds. Collectively, these results indicate that rodents tend to disperse and cache seeds with traits associated with greater energetic returns per unit handling time (Chang and Zhang 2014; Zhang, Shi, et al. 2016; Zhang, Yan, et al. 2016). We also found that SDE was consistently highest for *Q. variabilis* , intermediate for *Q. aliena* and lowest for *Q. glandulifera*, further indicating that *Q. variabilis* had a competitive advantage in seed dispersal over *Q. aliena* and *Q. glandulifera*.

In addition, rodents require more time to open seeds with thick shells, potentially increasing their exposure to predators during seed handling (e.g., Xiao et al. 2005; Rusch et al. 2013; Steele et al. 2015; Zhang, Shi, et al. 2016; Zhang, Yan, et al. 2016). Rodents may therefore remove seeds with thicker shells to safer locations for consumption or cache them for later use. In our study, *Q. variabilis* seeds had thick, hard shells, whereas those of *Q. glandulifera* were thinner and easier to open. This difference may explain why rodents removed and cached more *Q. variabilis* seeds but consumed more *Q. glandulifera* seeds, particularly in situ. These patterns suggest that the risk of predation to rodents during seed handling may also influence their seed‐removal and caching decisions (Zhang, Shi, et al. 2016; Zhang, Yan, et al. 2016).

Although dispersal distances did not differ significantly among the three seed species across years, there was a general tendency for *Q. variabilis* seeds to be transported farther before being eaten or cached than *Q. aliena* and *Q. glandulifera*. This pattern is consistent with predictions from the optimal caching space model, whereby rodents are expected to transport higher‐value food farther from seed release stations before caching it (Zhang, Shi, et al. 2016; Zhang, Yan, et al. 2016; Wang et al. 2021).

### 4.2 Annual and Seasonal Effects

Masting is an evolutionary strategy by which plants may increase their dispersal fitness under changing environmental conditions (e.g., Pearse et al. 2016; Bogdziewicz et al. 2018; Zhang et al. 2024). The three sympatric *Quercus* spp. in our study exhibited broadly synchronous masting and non‐masting among years (2016–2021), and seed‐harvesting rates differed significantly among years for all three *Quercus* spp. We found that seed harvest rates for the three oak species were significantly lower in years with higher seed abundance (PCSA). This pattern was most prominent in 2017, when PCSA peaked and seed harvest rates were relatively significantly the slowest observed throughout the study period. Conversely, seed harvest rates increased significantly in years with lower PCSA. This pattern may support the predator satiation hypothesis, whereby greater seed availability reduces the rate at which rodents remove or consume individual seeds (Yu et al. 2020; Wang et al. 2023).

The proportions of seeds eaten in situ (EIS), eaten after removal (EAR), and scatter‐ or larder‐cached (SLC) also differed among autumns from 2016 to 2021. The proportions of seeds consumed (EIS and EAR) were generally greater in years with higher PCSA, whereas the proportions cached (SLC) were generally lower, a pattern consistent with the predator dispersal hypothesis (e.g., Jansen et al. 2004; Yang et al. 2019). Together, these results support both the predator satiation and predator dispersal hypotheses, suggesting that the mechanisms underlying them can operate concurrently (Yang et al. 2019). Overall, SDE also tended to be higher in years with lower PCSA and lower in years with higher PCSA.

In spring, seed harvest rates for the three *Quercus* spp. also differed significantly among 2022–2024. However, harvest rates were substantially lower in spring than in autumn, most notably in 2023, when c. 15%, 50%, and 95% of *Q. variabilis* , *Q. aliena*, and *Q. glandulifera* seeds, respectively, remained in situ 50 days after release. One possible explanation is lower rodent abundance in spring following reduced breeding activity during winter, when food availability is limited (Hansson and Henttonen 1988). Moreover, when seeds of all three *Quercus* spp. were scarce in spring, rodents predominantly consumed them in situ and cached very few; no *Q. aliena* or *Q. glandulifera* seeds were cached in spring 2022 or 2023. These results suggest that seasonal variation can influence the seed predation and dispersal strategies of rodents.

## 5 Conclusion

Our findings indicate that seed traits, seed abundance, and seasonality jointly shape rodent‐mediated seed predation and dispersal among sympatric tree species. Rodents showed clear preferences among co‐occurring seeds with contrasting traits, with *Q. variabilis* having a competitive advantage in seed dispersal over *Q. aliena* and *Q. glandulifera*. This suggests that seed‐trait‐mediated selection by rodents may play an important role in modulating the effects of masting on rodent‐mediated seed predation and dispersal. Variation in seed predation and dispersal under different levels of seed abundance was consistent with both the predator satiation and predator dispersal hypotheses, suggesting that the mechanisms underlying these hypotheses can operate concurrently. Seasonal variation also influenced rodent seed predation and dispersal strategies: caching was more frequent in autumn, whereas consumption increased and caching declined in spring. Future studies should examine how seed traits and seed abundance regulate indirect seed‐seed interactions among sympatric tree species and thereby influence their coexistence.

## Author Contributions

**Jing Wang:** conceptualization (equal), data curation (equal), funding acquisition (equal), writing – review and editing (equal). **Ge Han:** data curation (equal). **Bo Zhang:** investigation (equal). **Ning Han:** investigation (equal). **Tuo Feng:** investigation (equal). **Jidong Zhao:** investigation (equal). **Xiaolei An:** investigation (equal). **Xiang Hou:** conceptualization (equal), writing – review and editing (equal). **Gang Chang:** conceptualization (equal), writing – review and editing (equal).

## Funding

This work was supported by the National Natural Science Foundation of China (32673261, 32500527, 31901085), Shaanxi Key Research and Development Program (2023‐YBSF‐295), the Shaanxi Natural Science Foundation (2025JC‐YBQN‐262, 2024JC‐YBMS‐188), Shaanxi Academy of Sciences Fundamental Research Program (2023K‐09), Science and Technology Program of Shaanxi Academy of Sciences (2024p‐06). Shangluo University Doctoral Research Development Fund (20SKY008).

## Conflicts of Interest

The authors declare no conflicts of interest.

## Supporting information


**Data S1:** Seed original data.

**Table S1:** Summary outputs of binomial generalized linear mixed‐effects models (GLMMs) examining differences in seed‐fate metrics among three oak species in autumn (2016–2021).
**Table S2:** Summary outputs of binomial generalized linear mixed‐effects models (GLMMs) examining differences in seed‐fate metrics among three oak species in spring (2022–2024).
**Table S3:** A Binomial GLMM outputs for inter‐annual variation of *Quercus variabilis* seed‐fate metrics in autumn (2016–2021).
**Table S4:** A Binomial GLMM outputs for inter‐annual variation of *Quercus aliena* seed‐fate metrics in autumn (2016–2021).
**Table S5:** A Binomial GLMM outputs for inter‐annual variation of *Quercus glandulifera* seed‐fate metrics in autumn (2016–2021).
**Table S6:** A Binomial GLMM outputs for inter‐annual variation of *Quercus variabilis* seed‐fate metrics in spring (2022–2024).
**Table S7:** A Binomial GLMM outputs for inter‐annual variation of *Quercus aliena* seed‐fate metrics in spring (2022–2024).
**Table S8:** A Binomial GLMM outputs for inter‐annual variation of *Quercus glandulifera* seed‐fate metrics in spring (2022–2024).

## Data Availability

Data from this study have been uploaded as Supporting Information for peer review. Once the article is published, these data will be made available to the public.
