# Supplementary material for: Trait‐Mediated Seed Predation and Dispersal Are Regulated by Seed Abundance for Sympatric Tree Species in the Qinling Mountains, China
Source: Ecol Evol. 2026 Oct 4;16(10):e74453. doi: 10.1002/ece3.74453 (PMC13635484; doi:10.1002/ece3.74453)
Supplement: Supplementary file 1 [file ECE3-16-e74453-s001.pdf]

Table S1 Summary outputs of binomial generalized linear mixed-effects models (GLMMs) examining differences in seed-fate metrics among three oak species in autumn (2016-2021)

Year	Response variable	Term	β coefficient	SE	95% CI-lower	95% CI-upper	P-value
2016	IIS/TS	Intercept	0.988	0.213	0.568	1.408	0
		species-1	-2.390	0.319	-3.020	-1.761	0
		species-2	-1.041	0.285	-1.603	-0.479	0
		species-3	0[a]	—	—	—	—
	EIS/TS	Intercept	-2.596	0.194	-2.978	-2.215	0
		species-1	-4.087	1.405	-6.855	-1.319	0.004
		species-2	-0.859	0.345	-1.538	-0.179	0.013
		species-3	0[a]	—	—	—	—
	EAR/TS	Intercept	-5.069	0.62	-6.291	-3.847	0
		species-1	2.057	0.662	0.752	3.361	0.002
		species-2	-0.000	0.877	-1.729	1.729	1
		species-3	0[a]	—	—	—	—
	SLC/TS	Intercept	-4.730	0.614	-5.939	-3.521	0
		species-1	2.497	0.643	1.23	3.765	0
		species-2	2.218	0.651	0.935	3.5	0.001
		species-3	0[a]	—	—	—	—
2017	IIS/TS	Intercept	2.022	0.3	1.431	2.614	0
		species-1	-1.280	0.364	-1.998	-0.562	0.001
		species-2	-0.771	0.38	-1.519	-0.023	0.043
		species-3	0[a]	—	—	—	—
	EIS/TS	Intercept	-2.881	0.292	-3.455	-2.306	0
		species-1	-1.112	0.571	-2.236	0.013	0.053
		species-2	-0.758	0.507	-1.756	0.24	0.136
		species-3	0[a]	—	—	—	—
	EAR/TS	Intercept	-3.958	0.524	-4.990	-2.925	0
		species-1	0.726	0.644	-0.543	1.995	0.261
		species-2	-0.270	0.795	-1.836	1.296	0.734
		species-3	0[a]	—	—	—	—
	SLC/TS	Intercept	-4.228	0.574	-5.359	-3.097	0
		species-1	1.017	0.674	-0.312	2.345	0.133
		species-2	0.538	0.725	-0.890	1.967	0.459
		species-3	0[a]	—	—	—	—
2018	IIS/TS	Intercept	-0.832	0.213	-1.252	-0.413	0
		species-1	-1.014	0.356	-1.716	-0.313	0.005
		species-2	-0.523	0.323	-1.159	0.113	0.107
		species-3	0[a]	—	—	—	—
	EIS/TS	Intercept	-3.085	0.19	-3.459	-2.710	0
		species-1	-1.984	0.529	-3.026	-0.943	0
		species-2	-0.349	0.293	-0.928	0.229	0.235
		species-3	0[a]	—	—	—	—
	EAR/TS	Intercept	-3.414	0.294	-3.993	-2.834	0
		species-1	0.883	0.356	0.181	1.584	0.014
		species-2	-1.120	0.584	-2.271	0.03	0.056
		species-3	0[a]	—	—	—	—
	SLC/TS	Intercept	-4.143	0.475	-5.079	-3.207	0
		species-1	0.949	0.564	-0.161	2.059	0.094
		species-2	1.145	0.55	0.061	2.228	0.038
		species-3	0[a]	—	—	—	—
2019	IIS/TS	Intercept	1.82	0.274	1.28	2.361	0
		species-1	-0.609	0.355	-1.308	0.091	0.088
		species-2	-0.354	0.367	-1.076	0.368	0.335
		species-3	0[a]	—	—	—	—
	EIS/TS	Intercept	-2.626	0.285	-3.188	-2.063	0
		species-1	-0.520	0.46	-1.426	0.385	0.259
		species-2	0.183	0.389	-0.583	0.949	0.638
		species-3	0[a]	—	—	—	—
	EAR/TS	Intercept	-5.582	0.989	-7.531	-3.633	0
		species-1	2.433	1.036	0.393	4.474	0.02
		species-2	0.407	1.278	-2.110	2.925	0.75

		species-3	0[a]	—	—	—	—
	SLC/TS	Intercept	-7.377	1.789	-10.901	-3.854	0
		species-1	3.312	1.822	-0.277	6.901	0.07
		species-2	2.202	1.886	-1.513	5.917	0.244
		species-3	0[a]	—	—	—	—
2020	IIS/TS	Intercept	1.45	0.24	0.977	1.923	0
		species-1	-2.920	0.341	-3.591	-2.249	0
		species-2	-1.365	0.305	-1.966	-0.764	0
		species-3	0[a]	—	—	—	—
	EIS/TS	Intercept	-5.582	0.483	-6.535	-4.630	0
		species-1	-1.795	1.277	-4.311	0.721	0.161
		species-2	0.513	0.612	-0.692	1.718	0.402
		species-3	0[a]	—	—	—	—
	EAR/TS	Intercept	-34.753	0.8	-36.330	-33.177	0
		species-1	30.796	0.84	29.142	32.45	0
		species-2	—	—	—	—	—
		species-3	0[a]	—	—	—	—
	SLC/TS	Intercept	-5.175	0.845	-6.839	-3.511	0
		species-1	3.039	0.869	1.326	4.751	0.001
		species-2	2.423	0.886	0.679	4.168	0.007
		species-3	0[a]	—	—	—	—
2021	IIS/TS	Intercept	0.72	0.177	0.372	1.068	0
		species-1	-2.279	0.281	-2.833	-1.725	0
		species-2	-0.953	0.243	-1.432	-0.474	0
		species-3	0[a]	—	—	—	—
	EIS/TS	Intercept	-2.646	0.14	-2.922	-2.369	0
		species-1	-1.776	0.351	-2.468	-1.083	0
		species-2	-0.532	0.227	-0.979	-0.086	0.02
		species-3	0[a]	—	—	—	—
	EAR/TS	Intercept	-5.293	0.741	-6.754	-3.833	0
		species-1	3.025	0.763	1.522	4.527	0
		species-2	1.301	0.839	-0.343	2.945	0.122
		species-3	0[a]	—	—	—	—
	SLC/TS	Intercept	-32.762	0.584	-33.912	-31.611	0
		species-1	28.442	0.722	27.019	29.864	0
		species-2	—	—	—	—	—
		species-3	0[a]	—	—	—	—

Table S2 Summary outputs of binomial generalized linear mixed-effects models (GLMMs) examining differences in seed-fate metrics among three oak species in spring (2022-2024)

Year	Response variable	Term	β coefficient	SE	95% CI-lower	95% CI-upper	P-value
2022	IIS/TS	Intercept	0.573	0.19	0.199	0.947	0.003
		species-1	-1.211	0.27	-1.743	-0.679	0
		species-2	0.381	0.278	-0.167	0.93	0.172
		species-3	0[a]	—	—	—	—
	EIS/TS	Intercept	-2.314	0.238	-2.783	-1.844	0
		species-1	-0.699	0.401	-1.489	0.092	0.083
		species-2	0.102	0.33	-0.548	0.753	0.757
		species-3	0[a]	—	—	—	—
	EAR/TS	Intercept	-4.369	0.351	-5.061	-3.678	0
		species-1	1.034	0.411	0.224	1.844	0.013
		species-2	-0.805	0.629	-2.044	0.433	0.202
		species-3	0[a]	—	—	—	—
	SLC/TS	Intercept	-33.759	0.47	-34.686	-32.832	0
		species-1	28.69	—	—	—	—
		species-2	0.007	791.032	-1558353.562	1558353.576	1
		species-3	0[a]	—	—	—	—
2023	IIS/TS	Intercept	1.774	0.289	1.205	2.344	0
		species-1	-1.166	0.359	-1.874	-0.459	0.001
		species-2	-0.548	0.378	-1.293	0.196	0.148
		species-3	0[a]	—	—	—	—
	EIS/TS	Intercept	-2.503	0.287	-3.068	-1.938	0
		species-1	0.069	0.4	-0.718	0.857	0.862
		species-2	-0.184	0.423	-1.016	0.649	0.664
		species-3	0[a]	—	—	—	—
	EAR/TS	Intercept	-3.860	0.406	-4.660	-3.061	0
		species-1	0.089	0.562	-1.019	1.197	0.874
		species-2	0.06	0.566	-1.055	1.175	0.916
		species-3	0[a]	—	—	—	—
	SLC/TS	Intercept	-35.752	0.065	-35.880	-35.624	0
		species-1	0	—	—	—	—
		species-2	0	—	—	—	—
		species-3	0[a]	—	—	—	—
2024	IIS/TS	Intercept	-0.307	0.186	-0.674	0.059	0.1
		species-1	-1.175	0.301	-1.768	-0.583	0
		species-2	-0.866	0.285	-1.428	-0.304	0.003
		species-3	0[a]	—	—	—	—
	EIS/TS	Intercept	-2.337	0.196	-2.722	-1.952	0
		species-1	-1.983	0.525	-3.017	-0.950	0
		species-2	-0.415	0.304	-1.015	0.185	0.174
		species-3	0[a]	—	—	—	—
	EAR/TS	Intercept	-4.185	0.294	-4.764	-3.605	0
		species-1	0.571	0.369	-0.157	1.299	0.123
		species-2	0.546	0.371	-0.185	1.277	0.142
		species-3	0[a]	—	—	—	—
	SLC/TS	Intercept	-7.377	1.761	-10.847	-3.907	0
		species-1	3.714	1.784	0.199	7.228	0.038
		species-2	2.782	1.816	-0.796	6.36	0.127
		species-3	0[a]	—	—	—	—

Table S3 A Binomial GLMM outputs for inter-annual variation of *Quercus variabilis* seed-fate metrics in autumn (2016-2021)

Response variable	Term	β coefficient	SE	95% CI-lower	95% CI-upper	P-value
IIS/TS	Intercept	-1.559	0.261	-2.073	-1.046	0
	year-2016	0.157	0.361	-0.552	0.866	0.663
	year-2017	2.302	0.336	1.64	2.963	0
	year-2018	-0.288	0.389	-1.053	0.478	0.461
	year-2019	2.771	0.352	2.08	3.463	0
	year-2020	0.089	0.365	-0.627	0.805	0.808
	year-2021	0[a]	—	—	—	—
EIS/TS	Intercept	-4.421	0.393	-5.194	-3.648	0
	year-2016	-2.262	1.269	-4.755	0.231	0.075
	year-2017	0.429	0.507	-0.567	1.425	0.397
	year-2018	-0.648	0.669	-1.961	0.666	0.333
	year-2019	1.275	0.448	0.395	2.156	0.005
	year-2020	-2.956	1.75	-6.394	0.483	0.092
	year-2021	0[a]	—	—	—	—
EAR/TS	Intercept	-2.269	0.254	-2.768	-1.769	0
	year-2016	-0.744	0.433	-1.594	0.107	0.087
	year-2017	-0.963	0.466	-1.878	-0.049	0.039
	year-2018	-0.262	0.383	-1.016	0.491	0.494
	year-2019	-0.880	0.453	-1.771	0.011	0.053
	year-2020	-1.689	0.602	-2.873	-0.505	0.005
	year-2021	0[a]	—	—	—	—
SLC/TS	Intercept	-4.320	0.61	-5.519	-3.121	0
	year-2016	2.088	0.654	0.803	3.372	0.001
	year-2017	1.109	0.708	-0.283	2.501	0.118
	year-2018	1.126	0.707	-0.263	2.514	0.112
	year-2019	0.255	0.814	-1.345	1.855	0.754
	year-2020	2.184	0.651	0.906	3.462	0.001
	year-2021	0[a]	—	—	—	—

Table S4 A Binomial GLMM outputs for inter-annual variation of *Quercus aliena* seed-fate metrics in autumn (2016-2021)

Response variable	Term	β coefficient	SE	95% CI-lower	95% CI-upper	P-value
IIS/TS	Intercept	-0.234	0.186	-0.599	0.131	0.209
	year-2016	0.181	0.262	-0.333	0.696	0.49
	year-2017	1.485	0.289	0.916	2.053	0
	year-2018	-1.122	0.294	-1.700	-0.543	0
	year-2019	1.7	0.301	1.109	2.29	0
	year-2020	0.319	0.262	-0.196	0.833	0.224
	year-2021	0[a]	—	—	—	—
EIS/TS	Intercept	-3.178	0.259	-3.687	-2.669	0
	year-2016	-0.277	0.393	-1.048	0.495	0.481
	year-2017	-0.460	0.413	-1.272	0.351	0.266
	year-2018	-0.256	0.39	-1.023	0.511	0.512
	year-2019	0.736	0.32	0.107	1.364	0.022
	year-2020	-1.891	0.695	-3.256	-0.526	0.007
	year-2021	0[a]	—	—	—	—
EAR/TS	Intercept	-3.992	0.312	-4.606	-3.379	0
	year-2016	-1.077	0.614	-2.283	0.129	0.08
	year-2017	-0.236	0.469	-1.157	0.686	0.616
	year-2018	-0.542	0.512	-1.548	0.465	0.291
	year-2019	-1.183	0.638	-2.437	0.072	0.065
	year-2020	-2.285	1.012	-4.274	-0.297	0.024
	year-2021	0[a]	—	—	—	—
SLC/TS	Intercept	-4.973	0.668	-6.286	-3.660	0
	year-2016	2.461	0.7	1.085	3.836	0
	year-2017	1.283	0.758	-0.206	2.773	0.091
	year-2018	1.975	0.717	0.566	3.383	0.006
	year-2019	-0.202	0.995	-2.158	1.754	0.839
	year-2020	2.221	0.707	0.832	3.611	0.002
	year-2021	0[a]	—	—	—	—

Table S5 A Binomial GLMM outputs for inter-annual variation of *Quercus glandulifera* seed-fate metrics in autumn (2016-2021)

Response variable	Term	β coefficient	SE	95% CI-lower	95% CI-upper	P-value
IIS/TS	Intercept	0.72	0.191	0.345	1.094	0
	year-2016	0.269	0.277	-0.276	0.813	0.333
	year-2017	1.303	0.337	0.64	1.966	0
	year-2018	-1.552	0.272	-2.087	-1.017	0
	year-2019	1.101	0.321	0.47	1.732	0.001
	year-2020	0.73	0.297	0.146	1.315	0.014
	year-2021	0[a]	—	—	—	—
EIS/TS	Intercept	-2.646	0.232	-3.101	-2.191	0
	year-2016	0.049	0.324	-0.588	0.686	0.879
	year-2017	-0.235	0.346	-0.914	0.445	0.498
	year-2018	-0.439	0.365	-1.155	0.278	0.23
	year-2019	0.02	0.326	-0.621	0.661	0.951
	year-2020	-2.936	0.971	-4.844	-1.029	0.003
	year-2021	0[a]	—	—	—	—
EAR/TS	Intercept	-5.293	0.576	-6.425	-4.162	0
	year-2016	0.224	0.773	-1.294	1.743	0.772
	year-2017	1.336	0.649	0.061	2.611	0.04
	year-2018	1.88	0.62	0.661	3.099	0.003
	year-2019	-0.289	0.879	-2.016	1.438	0.743
	year-2020	-30.458	—	—	—	—
	year-2021	0[a]	—	—	—	—
SLC/TS	Intercept	-33.817	0.597	-34.990	-32.643	0
	year-2016	29.087	0.766	27.581	30.592	0
	year-2017	29.589	0.705	28.203	30.975	0
	year-2018	29.673	0.697	28.303	31.044	0
	year-2019	26.439	1.884	22.737	30.142	0
	year-2020	28.642	—	—	—	—
	year-2021	0[a]	—	—	—	—

Table S6 A Binomial GLMM outputs for inter-annual variation of *Quercus variabilis* seed-fate metrics in spring (2022-2024)

Response variable	Term	β coefficient	SE	95% CI-lower	95% CI-upper	P-value
IIS/TS	Intercept	-1.483	0.246	-1.967	-0.998	0
	year-2022	0.845	0.318	0.219	1.47	0.008
	year-2023	2.091	0.317	1.466	2.715	0
	year-2024	0[a]	—	—	—	—
EIS/TS	Intercept	-4.320	0.517	-5.338	-3.302	0
	year-2022	1.308	0.587	0.152	2.464	0.027
	year-2023	1.886	0.56	0.783	2.989	0.001
	year-2024	0[a]	—	—	—	—
EAR/TS	Intercept	-3.613	0.311	-4.226	-3.001	0
	year-2022	0.278	0.414	-0.537	1.093	0.502
	year-2023	-0.158	0.457	-1.059	0.743	0.73
	year-2024	0[a]	—	—	—	—
SLC/TS	Intercept	-3.664	0.329	-4.312	-3.015	0
	year-2022	-1.405	0.73	-2.844	0.034	0.056
	year-2023	-32.088	—	—	—	—
	year-2024	0[a]	—	—	—	—

Table S7 A Binomial GLMM outputs for inter-annual variation of *Quercus aliena* seed-fate metrics in spring (2022-2024)

Response variable	Term	β coefficient	SE	95% CI-lower	95% CI-upper	P-value
IIS/TS	Intercept	-1.173	0.223	-1.612	-0.734	0
	year-2022	2.127	0.307	1.522	2.732	0
	year-2023	2.399	0.317	1.774	3.024	0
	year-2024	0[a]	—	—	—	—
EIS/TS	Intercept	-2.752	0.296	-3.334	-2.169	0
	year-2022	0.54	0.378	-0.204	1.284	0.154
	year-2023	0.065	0.412	-0.747	0.877	0.876
	year-2024	0[a]	—	—	—	—
EAR/TS	Intercept	-3.638	0.247	-4.125	-3.151	0
	year-2022	-1.537	0.578	-2.675	-0.398	0.008
	year-2023	-0.162	0.364	-0.879	0.555	0.657
	year-2024	0[a]	—	—	—	—
SLC/TS	Intercept	-4.595	0.216	-5.021	-4.169	0
	year-2022	-31.157	—	—	—	—
	year-2023	-31.157	—	—	—	—
	year-2024	0[a]	—	—	—	—

Table S8 A Binomial GLMM outputs for inter-annual variation of *Quercus glandulifera* seed-fate metrics in spring (2022-2024)

Response variable	Term	β coefficient	SE	95% CI-lower	95% CI-upper	P-value
IIS/TS	Intercept	-0.307	0.192	-0.687	0.072	0.112
	year-2022	0.88	0.276	0.336	1.424	0.002
	year-2023	2.082	0.332	1.428	2.735	0
	year-2024	0[a]	—	—	—	—
EIS/TS	Intercept	-2.337	0.252	-2.833	-1.841	0
	year-2022	0.023	0.354	-0.675	0.721	0.948
	year-2023	-0.167	0.369	-0.894	0.56	0.652
	year-2024	0[a]	—	—	—	—
EAR/TS	Intercept	-4.185	0.38	-4.933	-3.436	0
	year-2022	-0.185	0.563	-1.294	0.924	0.743
	year-2023	0.324	0.5	-0.660	1.309	0.517
	year-2024	0[a]	—	—	—	—
SLC/TS	Intercept	-7.377	0.578	-8.515	-6.239	0
	year-2022	-28.375	—	—	—	—
	year-2023	-28.375	—	—	—	—
	year-2024	0[a]	—	—	—	—
